# Development of Quantitative Proteomics Using iTRAQ Based on the Immunological Response of *Galleria mellonella* Larvae Challenged with *Fusarium oxysporum* Microconidia

**DOI:** 10.1371/journal.pone.0112179

**Published:** 2014-11-07

**Authors:** Amalia Muñoz-Gómez, Mauricio Corredor, Alfonso Benítez-Páez, Carlos Peláez

**Affiliations:** 1 Grupo Interdisciplinario de Estudios Moleculares (GIEM), Instituto de Química, Universidad de Antioquia, Medellín, Antioquia, Colombia; 2 Genetic and Biochemistry of Microorganisms group (GEBIOMIC), Instituto de Biología, Universidad de Antioquia, Medellín, Antioquia, Colombia; 3 Bioinformatic Analysis Group (GABi), Centro de Investigación y Desarrollo en Biotecnología, CIDBIO, Bogotá, Distrito Capital, Colombia; Warren Alpert Medical School of Brown University, United States of America

## Abstract

*Galleria mellonella* has emerged as a potential invertebrate model for scrutinizing innate immunity. Larvae are easy to handle in host-pathogen assays. We undertook proteomics research in order to understand immune response in a heterologous host when challenged with microconidia of *Fusarium oxysporum*. The aim of this study was to investigate hemolymph proteins that were differentially expressed between control and immunized larvae sets, tested with *F. oxysporum* at two temperatures. The iTRAQ approach allowed us to observe the effects of immune challenges in a lucid and robust manner, identifying more than 50 proteins, 17 of them probably involved in the immune response. Changes in protein expression were statistically significant, especially when temperature was increased because this was notoriously affected by *F. oxysporum* 10^4^ or 10^6^ microconidia/mL. Some proteins were up-regulated upon immune fungal microconidia challenge when temperature changed from 25 to 37°C. After analysis of identified proteins by bioinformatics and meta-analysis, results revealed that they were involved in transport, immune response, storage, oxide-reduction and catabolism: 20 from *G. mellonella*, 20 from the Lepidoptera species and 19 spread across bacteria, protista, fungi and animal species. Among these, 13 proteins and 2 peptides were examined for their immune expression, and the hypothetical 3D structures of 2 well-known proteins, unannotated for *G. mellonella*, i.e., actin and CREBP, were resolved using peptides matched with *Bombyx mori* and *Danaus plexippus*, respectively. The main conclusion in this study was that iTRAQ tool constitutes a consistent method to detect proteins associated with the innate immune system of *G. mellonella* in response to infection caused by *F. oxysporum*. In addition, iTRAQ was a reliable quantitative proteomic approach to detect and quantify the expression levels of immune system proteins and peptides, in particular, it was found that 10^4^ microconidia/mL at 37°C over expressed many more proteins than other treatments.

## Introduction

*Galleria mellonella* larvae offer considerable advantages as an infection and virulence model because they are simple to handle and can be studied in large numbers while carrying out cost-effective experiments [1]. Most importantly, and unlike many other invertebrate models such as *Drosophila melanogaster* and Caenorhabditis elegans species, experiments can be performed at 37°C, an optimal temperature for the vast majority of human pathogens [2]. The remarkable advantages of this model also draw upon the innate immune response of *G. mellonella* larvae, which share a high degree of homology with mammalian organisms [3].

Furthermore, *G. mellonella* has been widely used as a heterologous host for several fungal pathogens, including *Candida albicans*, *Cryptococcus neoformans* and *Aspergillus fumigatus* [4]. A positive correlation has been observed in pathogenicity using these yeasts or fungi since this insect model is comparable to mammalian models [5]. For example, *C. neoformans* can proliferate in the *G. mellonella* hemocoel, leading to the eventual death of the caterpillar. Therefore, it is unquestionable that *G. mellonella* should emerge as a model of host defense against fungal infection [6].

The hemocytes in the cellular hemolymph produce a robust oxidative burst, which performs similarly to phagocytic cells in response to bacterial infections [7]. This feature, among other conserved aspects of the anti-microbial process, might explain the positive correlation between data obtained from *Galleria* and mice infections [8] with either prokaryotic or eukaryotic pathogens. Therefore, *G. mellonella* emerges as a model that has since been successfully applied to assess the virulence in a variety of bacteria, including the Burkholderia cepacia complex [9], Enterococcus faecium [10], *Francisella tularensis* [11], Legionella pneumophila [12], Listeria monocytogenes [13], Pseudomona aeruginosa [8], [14] and various corynebacteria [15]. Additionally, virulence in *G. mellonella* has been assessed using various fungi, as mentioned above, A. fumigatus [16], C. albicans [17] and C. neoformans [6] and evaluated with some viruses, such as baculovirus [18] and parvovirus [19].

*Fusarium oxysporum* is a trans-kingdom pathogen and is well known for producing harmful secondary metabolites, called mycotoxins, that cause several diseases in humans, animals, insects, and even plants [20], [21]. This mold is the causal agent of vascular wilt disease, which affects a wide range of plant species as tomato, but also can produce disseminated infections in human beings and immunodepressed mice. The *Fusarium* species are an ideal model for a comparative analysis of fungal virulence in plant and animal hosts because they are capable of manifesting a multitude of clinical infections [20]. Few studies have been developed to use the larvae of *G. mellonella* as a heterologous host for fusaria. *G. mellonella* is able to resist high concentrations of *F. oxysporum* microconidia. When conidia are injected into the hemocoel of this Lepidopteran system, both clinical and environmental isolates of the fungus are able to kill the larvae at 37°C, although the killing occurs rapidly when incubated at 30°C [21].

Regarding the immune response of insects against fungal infections, the insect hemolymph plays a critical role, acting as a means of transporting cells, proteins-peptides, oxygen, hormones, nutrients, and metabolites associated with the immune system [22], [23]. Immunological studies of insects represent an important challenge to understand the molecular mechanisms of the innate immune response in the animal kingdom. Moreover, the innate immune system is well known as the primary defense against pathogen or competitors' attacks because it is widespread in all three major kingdoms of life [24].

Multiplexed high-throughput proteomics strategies provide an integrated and broader view of biological regulatory networks and pathways [25]. Protein activity is a crucial task that depends on the interactions and modifications of antagonistic and synergistic proteins [25]. iTRAQ is suitable for exploratory molecular studies of the pathogenic mechanisms and pathophysiology of diseases [26].

This study likely represents one of the first reports that uses iTRAQ to detect *G. mellonella* proteins after a fungal challenge because the main obstacle to the development of a computational approach with iTRAQ is the unavailability of *Galleria*'s genome, which makes protein identification problematic. Our proposal in this experimental and computational biology work was to apply an alternative approach, matching up the isolated iTRAQ peptides with the few *G. mellonella* proteins in databases and even with those from related butterflies or other organisms that have been previously reported in databases. In this work, we implemented a proteomic iTRAQ approach to detect and quantify protein expression patterns in *G. mellonella* hemolymph following challenge with a sub-lethal infection of *F. oxysporum* microconidia in order to induce a response of the innate immune system.

## Materials and Methods

### Maintenance of biological samples

Sixth-instar larvae of the wax moth *G. mellonella* were reared in our laboratory and fed an artificial diet (5.54% dry yeast, 22.17% wheat bran, 11.09% yellow corn flour, 11.09% powdered milk, 5.54% wheat germ, 17.65% honey, 22.70% glycerol, 3.88% beeswax and 0.033% formaldehyde solution). Larvae with a body weight between 230 and 330 mg were used. Caterpillars were harvested for treatments with the best quality control (color, size and vigor). Additionally, a strain of *F. oxysporum* (Foxy-GIEM isolated from soil) was grown in PDA (Oxoid, Ltd., Basingstoke, England) and maintained at 25°C for 14 days at intervals of light and darkness (16 h light/8 h dark).

### *F. oxysporum* microconidia preparation

A surfactant solution of 0.1% Tween-80 was added to isolate the conidia from the mycelium, using Drigalski spatula for scraping. The resulting suspension was filtered through sterile gauze and collected in a sterilized screw-cap tube. The volume was increased to 10 mL with 0.1% Tween-80. The microconidia suspension was vortexed three times for 20 seconds each. The concentration of the suspension was determined with the Neubauer–improved counting chamber (Paul Marienfeld GmbH & Co. KG, Lauda-Königshofen, Germany) and adjusted to 1×10^4^–1×10^8^ microconidia/mL.

### Larval survival test and lethal concentration of microconidia

*G. mellonella* larvae were arranged into 10 groups, randomly selected during the last instar of the larval development and inoculated with conidia (1×10^4^, 1×10^5^, 1×10^6^, 1×10^7^ and 1×10^8^ microconidia/mL) by injecting 20 µL of suspension into the hemocoel through the last pro-leg [27]. Before injection, the area was disinfected with an alcohol swab (70%). A 1 mL disposable syringe was used with a 31G×8.0 mm needle (BD Ultra-Fine, Becton Dickinson, Franklin Lakes, NJ, USA). Larvae were stored in the dark and incubated in Petri dishes at 25 and 37°C. The experiment was prepared with two control groups: the first group represented the control injection process, regarding the physical trauma into hemocoel of larvae [6] inoculated with 20 µL of 0.1% Tween-80, and the second control group received no injection. Mortality was assessed after 48 hours based on the brown color of the cuticle (melanization) and the absence of movement in response to stimulation. All experiments were performed in triplicate. Each experiment was classified as sublethal microconidia concentrations when approximately 80–100% of the larvae survived.

### Statistics for the survival treatment of *G. mellonella*

To check if any assumption has been violated on the statistically significant difference between microconidia treatments and temperature, the hypothesis for testing the difference in proportions was established. To assess whether there was a relation of statistical significance between treatment and temperature. The hypothesis for testing a difference in the means of survival when temperature changed was performed for each microconidia concentration (1×10^4^, 1×10^5^, 1×10^6^ microconidia/mL and means of survival at 25 and 37°C). The p-values were calculated using R software (α≤0.5).

### Infection of larvae with microconidial sublethal concentrations

*G. mellonella* larvae, in groups of 10, were inoculated with conidia by injecting 20 µL of the suspension into the hemocoel through the last pro-leg with the sub-lethal concentrations selected, 1×10^4^ and 1×10^6^ microconidia/mL [27]. Two groups of 10 larvae were arranged in triplicate; one set was incubated at 25°C, and the other set was incubated at 37°C in Petri dishes after being kept in the dark for 48 hours. To ensure that larvae did not die by different treatments such as a single puncture injection or incubation at 37°C or with 20 µL of 0.1% Tween-80 surfactant solution, these processes were included as part of the treatments as controls [6]. The mortality rate was recorded 2 days post-injection. Mortality was assessed based on the brown cuticle coloration (melanization) and the absence of movement in response to stimulation. All experiments were performed in triplicate. The same statistical analysis was performed to know if there was a statistical significance. The same hypothesis for testing the difference in the means of survival was used when temperature changed for each microconidia concentration (1×10^4^ and 1×10^6^ microconidia/mL and means of survival at at 25 and 37°C). The p-values were calculated using R software (α≤0.5).

### Collection and preparation of cell-free hemolymph

Prior to hemolymph collection, insects were anesthetized by chilling for 5 min at 8°C, and the abdominal surface was disinfected with a 70% (v/v) ethanol solution. Hemolymph samples were obtained by puncturing the larval abdomen with a sterile insulin needle. The outflowing hemolymph was immediately collected into sterile, chilled Eppendorf tubes containing 0.150 mM phenylthiourea [28] to prevent melanization. The cell-free hemolymph was obtained by centrifugation at 1606 *g* for 5 min and then for 15 min at 4°C to pellet the cell debris. The pooled supernatants were stored at −20°C [29].

### Preparation and quantification of proteins

The protein concentration was determined with the Bradford method using bovine serum albumin (BSA) as a standard [29], [30]. The Quick Start Bradford protein assay kit (Bio-Rad Laboratories, Inc. USA) was used following the manufacturer's instructions.

### Identification and characterization of peptides by iTRAQ

The sample preparation for LC/MS/MS first used 100 µg of protein from each hemolymph sample, challenged or unchallenged with microconidia. The samples were processed using the protocol of Applied Biosystems iTRAQ 8-plex [31].

### Labeling of free-cell hemolymph sample

The hemolymph sample from larvae challenged with 1×10^4^ microconidia/mL of *F. oxysporum* and incubated at 25°C for 48 h was labeled as iTRAQ-113. The hemolymph of larvae injected with 0.1% Tween-80 and incubated at 25°C for 48 h was labeled as iTRAQ-114. The hemolymph sample from larvae challenged with 1×10^4^ microconidia/mL and incubated at 37°C for 48 h was labeled as iTRAQ-115. The hemolymph from larvae without any injection and incubated at 25°C for 48 h was labeled as iTRAQ-116. The sample from larvae challenged with 1×10^6^ microconidia/mL and incubated at 37°C for 48 h was labeled as iTRAQ-117. The sample from larvae injected with 0.1% Tween-80 and incubated at 37°C for 48 h was labeled as iTRAQ-118. Treatments are summarized in table 1.

**Table 1:** Treatments with microconidia concentrations and temperatures.

iTRAQ 8-plex tags	Microconidia concentration and temperatures	Volume recovered
113	Hemolymph challenge with *F.oxysporum* 10^4^ microconidia/mL at 25°C	6 mL
114	Hemolymph injected only with Tween-80 (0.1% v/v) at 25°C	6 mL
115	Hemolymph challenge with *F.oxysporum* 10^4^ microconidia/mL at 37°C	6 mL
116	Hemolymph untreated at 25°C	6 mL
117	Hemolymph challenge with *F.oxysporum* 10^6^ microconidia/mL at 37°C	6 mL
118	Hemolymph injected only with Tween-80 (0.1% v/v) at 37°C	6 mL

Sample tags for iTRAQ analysis. For each treatment or pool around 6 mL were recovered. The protein concentration was calculated by Bradford assay.

### Triple TOF mass spectrometry

The multiplexed isobaric chemical tagging reaction was elaborated in the YPED proteomics laboratory, Yale Cancer Center Mass Spectrometry Resource & W.M. Keck Foundation Biotechnology Resource Laboratory. Peptides were separated on a Waters nanoACQUITY system (75 µm x 150 mm BEH C18 eluted at 500 nL/min) via MS analysis on an AB Sciex 5600 Triple TOF mass spectrometer. The iTRAQ allowed the multiplexing of two to six protein samples and produced identical MS/MS sequencing ions for all six versions of the same derivatized tryptic peptide. Quantitation was achieved by comparing the peak areas and the resultant peak ratios for the six MS/MS reporter ions, which range from 113 to 118 Da (119 and 121 were not used for this experiment). The mass spectrometric analysis was visualized using the Peakview software and quantified with ProteinPilot (AB Sciex, MA, USA).

### Statistics and bioinformatics analyses

The LC/MS/MS raw data were analyzed using the Mascot Distiller software, version 2.2 (Matrix Science, United Kingdom), to match the peptides with a protein database using the first default parameter. The protein ratio type was set to median, the normalization method was median ratio, no outlier removal was chosen and the peptide threshold was set to at least homology. Mascot search engine with ProteinPilot software was utilized. The search for proteins was performed using the non-redundant NCBInr_20121109 (National Center for Biotechnology Information) database. Proteins matching two or more peptides were considered a positive identification, but trypsin peptides, the enzyme that digests proteins, were excluded from analysis. Protein concentrations were determined using the ProteinPilot software, version 4.0 (AB Sciex, MA, USA) to infer concentrations based on the amounts of peptides assigned. ProteinPilot software, which employs the Paragon search algorithm, was used for peptide matching, protein identification and relative protein quantitation. The ProteinPilot Descriptive Statistics Template (PDST) tool automatically generates a wealth of important information from data-intensive proteomics experiments. Protein expression ratios were calculated from the pair-wise comparison of two iTRAQ channels. For each ratio, the iTRAQ peak area was corrected for Observed Bias Correlation. The general level of confidence was 66% for the protein hits. The percent of confidence was expressed in ProtScore units. The levels of confidence were 99% (2.0), 95% (1.3), 90% (1.0) and 66% (0.47), and the ProtScore units are presented in parentheses. The results file data was stored in YPED proteomics database, School of Medicine, WM Keck foundation, Yale University. The P-value (Probability that the deviation from unity is by chance) of ratios was calculated (biased and non-biased). Identified Lepidoptera proteins were compared with *G. mellonella* peptides using pairwise alignment with the Blast algorithm version BLAST 2.2.29 [32] and multiple alignments with Muscle version 3.2 [33]. Protein data were analyzed in Uniprot, Pfam, Gene Ontology and KEGG to identify the biological processes associated with immunological functions.

### Validation of iTRAQ protein expression test with RT-PCR

In order to validate protein expression detected by iTRAQ, we compared these results with Real-Time-PCR (RT-PCR). Four larvae were previously treated with 10^6^ microconidia/mL and four were untreated, both at 37°C. RNA was extracted from hemolymph and cDNA synthesis was elaborated with Fermentas Thermo Scientific kit, following manufacturer's instructions. Three genes were selected randomly from iTRAQ list. RT-PCRs were developed with specific primers and the reactions were set on the Applied Biosystem device following classic quantitative PCR (qPCR) protocol. The genes selected were: serpin, cationic protein 8p and 26 kDa ferritin subunit (see table S4). The housekeeping genes were Ribosomal protein S7e, lipocalin and fungal protease inhibitor. The RT-PCR amplification data were compared with iTRAQ protein expression data using Log2 value of fold-changes. Both iTRAQ and RT-PCR were correlated statistically. The chi-square goodness of fit analysis was performed to determine whether there was correspondence between the iTRAQ and qPCR data obtained for the indicated proteins. The p-values greater than 0.05 indicate that both sets of data match.

### Secondary and tertiary structure of proteins

Peptides of two unannotated proteins for *G. mellonella* that had an adequate peptide coverage (more than 50%) were used to be aligned with other protein sequences. Peptides of each protein were reanalyzed by Blast version 2.2.29 [32] and the alignments were elaborated using the Muscle version 3.2 [33] and ClustalW version 2.0 algorithms [34]. Secondary and tertiary structures were defined using Phyre version 2.0 and i-Tasser version 3.0 servers on line and the standalone package. The files generated were visualized in UCSF Chimera version 1.8. The mutated amino acids were used to generate tertiary structures that were aligned with the PBD structure database. Additionally, the tertiary structures were aligned using structural comparison and 3D matching in Chimera. Hypothetical structures were analyzed and reported [35], [36].

## Results

### Survivor assay and selection of non-lethal dose from microconidia

**Figure 1:**
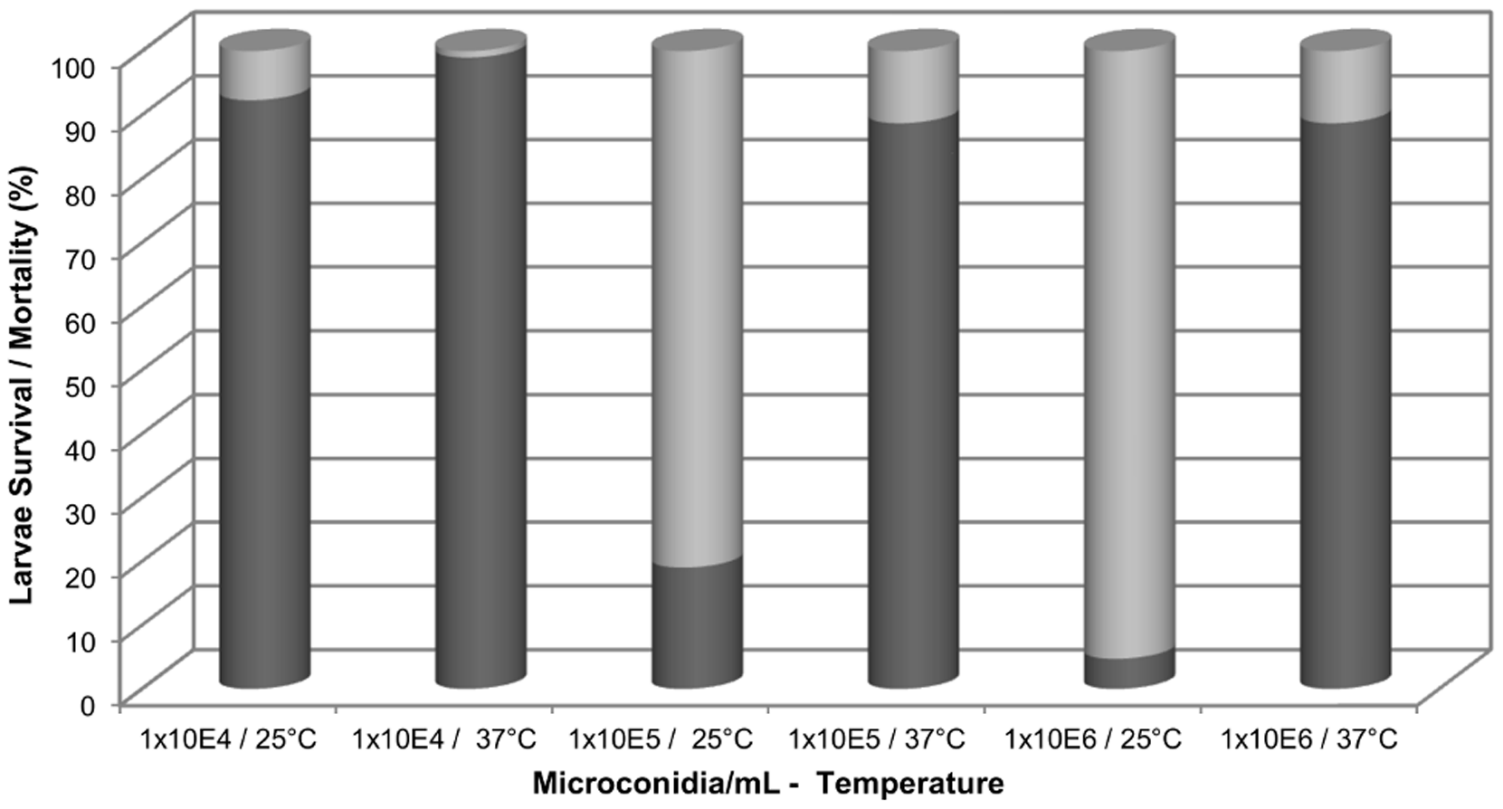
Survival/mortality percentage of larvae. Bars show *G. mellonella* larvae survivors in dark gray and dead larvae in light gray after being infected with 1×10^4^, 1×10^5^ and 1×10^6^ microconidia/mL of *F. oxysporum* at 25 or 37°C. Comparing the results, larval survival was higher at 37 than at 25°C.

To characterize the interaction between *G. mellonella* and *F. oxysporum*, we first reproduced the infection model to investigate the biological processes, such as protein transport, defense and nodulation, protein and lipid condensation and, finally, insect immune response during fungal infection. For this purpose, we inoculated larvae with different microconidia doses at two different temperatures. We noted that larvae treated with 10^4^, 10^5^, 10^6^ microconidia/mL at 25°C survived, whereas the mortality increased when microconidia concentration was higher (figure 1). The difference in means test obtained a p-value <0.01 for all treatments (10^4^, 10^5^, 10^6^ microconidia/mL concentrations), indicating that the survival mean of each treatment differs with temperature. In other words, mortality and survival are correlated and the same correlation would be obtained working with mortality rate (Data in Table S2). The experiment was repeated with new larvae with 10^4^ and 10^6^ microconidia/mL using the same statistical treatment. Once again, similar results were obtained (data in table S2). That is, at a fixed concentration, the average surviving larvae at 25°C is different from mean survival of larvae at 37°C, the latter being higher for both concentrations (Data in Table S3).

The best larvae survival rate throughout the study (∼99%) was observed at the 10^4^ microconidia/mL concentration at 37°C (figure 1). An increase in the survival was also observed at 37°C and was greater than at 25°C after treatments with 10^5^ and 10^6^ microconidia/mL. When concentrations of 10^7^ or 10^8^ microconidia/mL were used, every larva died in both temperatures. Using 10^6^ microconidia/mL at 25°C produced a low number of survivors and data were not included in figure 1.

To identify the proteins obtained by larvae challenged with *F. oxysporum*, we selected two microconidia concentrations where 10^4^ microconidia/mL produced a lower mortality than 10^6^ microconidia/mL did. This last concentration, despite the mortality, allowed us to retrieve a suitable number of larvae survivors for subsequent experiments.

### Protein identification inside larval hemolymph infected with fungi

A total of 374 peptides corresponding to 59 different proteins were identified in each treatment made by iTRAQ 8 plex. Regarding Lepidoptera, iTRAQ 8 plex identified 351 peptides dispersed among 40 proteins (table 2). Among these, 47 proteins were identified with a level of confidence ≥99%, and 54 proteins were identified with a confidence level ≥95%. From this set of proteins identified, 20 correspond to *G. mellonella* and 20 to the following Lepidoptera order: *Danaus plexippus* (4), *Bombyx mori* (3), *Corcyra cephalonica* (2), *Hyphantria cunea* (1), *Papilio polytes* (1), *Manduca sexta* (1), *Sesamia nonagrioides* (1), *Pieris rapae* (1), *Arcte modesta* (1), *Chilo suppressalis* (1), *Papilio xuthus* (1), *Samia ricini* (1), *Choristoneura fumiferana* (1), *Spodoptera exigua* (1) (table 2). The remaining proteins were identified as belonging to other Insecta orders, other invertebrates, bacteria, protozoan and fungi (table S1). Notably, more than 350 peptides were assigned to Lepidopteran proteins (table 2).

**Table 2:** List of proteins identified by iTRAQ, from the highest to the lowest protein score.

Accession	Protein name from *G. mellonella*	Peptides	Accession	Protein name from other Lepidotera species	Peptides
AAT76806	Apolipophorin	89	EHJ68005	apolipophorins, *Danaus plexippus*	2
AAA74229	Arylphorin	48	AGR44824	actin, *Bombyx mori*	7
AAQ63970	transferrin precursor	31	ADA84299	hexamerin receptor, *Corcyra cephalonica*	6
AAA19801	Hexamerin	22	Q6VU70	apolipophorin-3 precursor, *Hyphantria cunea*	1
P80703	apolipophorin-3; precursor	20	ADR64702	antennal esterase CXE5, *Spodoptera exigua*	1
AAQ75026	prophenoloxidase subunit 2	11	BAK82317	apolipophorin precursor, *Bombyx mori*	2
P83632	27 kDa hemolymph protein; precursor	12	EHJ79039	cellular retinoic acid binding protein, *Danaus plexippus*	3
ACU09501	Hemolin	21	BAM18997	imaginal disc growth factor 4, *Papilio polytes*	4
AAK64363	prophenoloxidase	14	Q25490	apolipophorin-2; precursor, *Manduca sexta*	3
AAN06604	juvenile hormone binding protein	8	NP_001037386	glyceraldehyde-3-phosphate dehydrogenase, *Bombix mori*	1
P82174	lysozyme; 1,4-beta-N-acetylmuramidase	6	AAG44959	hexamerin 2, *Corcyra cephalonica*	1
CAK22401	beta-1,3-glucan recognition protein precursor	1	AAY26453	moderately methionine rich storage protein, *Sesamia nonagrioides*	2
ADI87454	cationic protein 8 precursor	5	ACZ68116	masquerade-like serine proteinase, *Pieris rapae*	3
ABG91580	larval hemolymph protein	5	EHJ65451	moderately methionine rich storage protein, *Danaus plexippus*	2
AAL47694	32 kDa ferritin subunit	4	EHJ75277	serpin 1, *Danaus plexippus*	1
ACQ99193	proline-rich protein	2	ADX62478	isocitrate dehydrogenase, *Arcte modesta*	1
AAG41120	26kDa ferritin subunit	3	AEW46856	seminal fluid protein, *Chilo suppressalis*	1
P85210	cecropin-D-like peptide	1	BAM19609	peptidoglycan recognition protein, *Papilio xuthus*	2
P85216	anionic antimicrobial peptide 2	1	BAF42698	hemolymph storage protein 1, *Samia ricini*	1
ADK26057	kunitz-type protease inhibitor precursor	1	AAC35429	diapause associated protein 2, *Choristoneura fumiferana*	2

Left Column shows 20 proteins from *G. mellonella*. Right Column displays 20 proteins from other Lepidoptera species, which are not identified with some sequence of *G. mellonella* protein reported in database. They matched other butterfly species.

**Figure 2:**
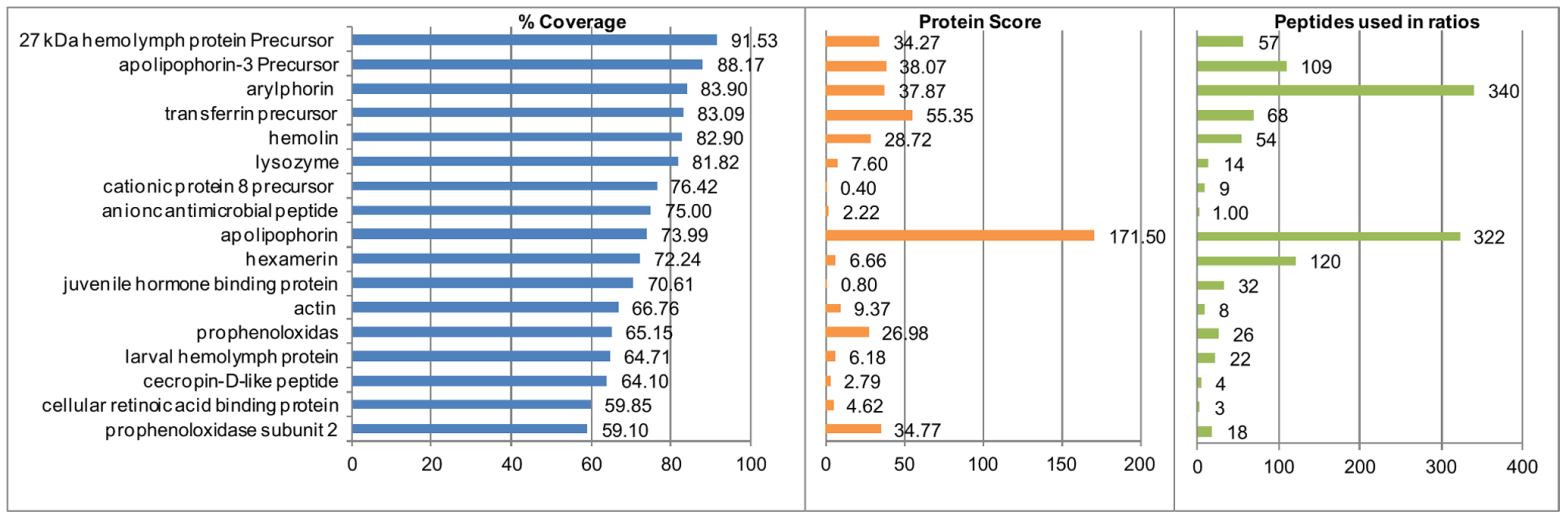
Data analysis of peptides from 17 proteins selected. Peptide percentage coverage is represented by the blue bar plot at the left; the protein score by the orange bar plot in the middle; and the peptides used in the ratios by the green bar plot to the right. The peptide ratios and protein scores were calculated using the ProteinPilot software. Selected proteins had more than 58% coverage.

Among the 59 proteins identified by iTRAQ, 17 showed good coverage with two or more peptides, as summarized in figure 2. Among these, the minimal peptide coverage was 59.85, corresponding to a cellular retinoic acid binding protein with 3 peptides; the maximum coverage was arylphorin with 340 labeled peptides detected (peptides in plot ratios, figure 2), corresponding to 48 different peptides with 83.9 coverage (figure 2). We selected those 17 proteins, not only for their coverage, but also for their excellent detection level in LC/MS/MS with different treatments. Of those proteins, 15 correspond to *G. mellonella*, 1 to *B. mori* (actin) and 1 to *D. plexippus* (cellular retinoic acid binding protein, belongs to the lipocalin family). Concerning the 20 Lepidopteran proteins, 18 showed peptide coverages lower than 50% (table 2).

### Protein expression in challenged larval hemolymph

The relationship between treatment ratios was compared to determine expression of each protein. Biased and unbiased statistical analyses were developed to establish the best differences. Table S4 displays the data ratios (non-biased) between the values of the different treatments and its p-values for the hypothesis test that the deviation from unity is by chance. The p-values lower than 0.05 indicate significant differences among the values of the compared treatments for 113 denominator (channel). From 17 proteins analyzed, 2 had only one peptide (non-significant, without p-value). For the other 15, 4 significant p-values for 115∶113 ratio, 8 significant p-values for 116∶113 ratio and 12 significant p-values for 117∶113 ratio were observed (Data in table S5).

**Figure 3:**
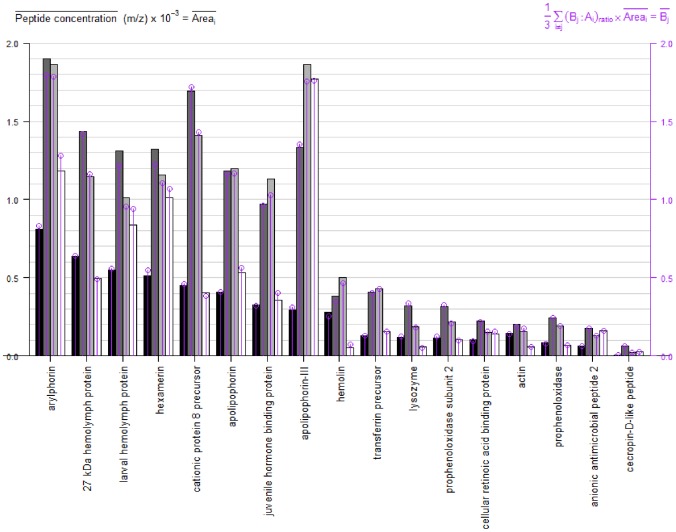
Results of protein expression of 17 proteins. The plot represents a comparative data expression level of the quantitative peptide concentration from 0 to 2000 units from the selected main results of 17 proteins identified by iTRAQ using ProteinPilot. Black bars correspond to 10^4^ microconidia/mL at 25°C, dark gray bars correspond to 10^6^ microconidia/mL at 37°C, light gray bars correspond to 10^4^ microconidia/mL at 37°C and white bars correspond to controls at 25°C untreated. Left y-axis axis is the average of the sum of the peptide areas, m/z (Area), as represented in black, gray and white bars. Right y-axis (purple lines) represent the average of the sum of the iTRAQ ratios per the mean of the sum from the peptide areas, m/z or Vp.

We analyzed 17 proteins inside each treatment that were affected (up- or down-regulated) by a pathogenic infection with *F. oxysporum* microconidia (10^4^ or 10^6^ microconidia/mL) at two temperatures (25 or 37°C). The relevant protein expression results from those proteins are summarized in figure 3, though only the three notable treatments (larvae with 10^4^ microconidia/mL at 25 or 37°C and larvae with 10^6^ microconidia/mL at 37°C) with controls (larvae without microconidia injection at 25°C) are shown in figure 3.

When the larvae were inoculated with 10^4^ microconidia/mL at 25°C, some proteins lowered their concentrations compared with untreated larvae at 25°C; the proteins that were down-regulated included: arylphorin, hexamerin, larval hemolymph, apolipophorin, apolipophorin 3, juvenile hormone binding protein, transferrin precursor, cellular retinoic acid binding proteins and anionic antimicrobial peptides. Conversely, the following proteins were up-regulated under the same conditions compared with untreated larvae at 25°C: 27 kDa hemolymph, cationic 8 precursor, hemolin, transferrin precursor, prophenoloxidase subunit 2, lysozime and actin. The level of cecropin D-like peptide was evidently low and was down-regulated in 10^4^ microconidia/mL at 25°C; this concentration was the lowest among the 17 proteins (figure 3).

Concerning protein expression with 10^4^ microconidia/mL treatment at 37°C, compared with 10^4^ microconidia/mL at 25°C or no treatment at 25°C, the results were quite different. With the exception of apolipophorin 3, all of the proteins were up-regulated with respect to the control (see untreated at 25°C, apolipophorin 3, figure 3) and showed higher protein concentrations. However, more interestingly, in each case of treatment with 10^4^ microconidia/mL at 37°C, the protein concentration was higher than 10^4^ microconidia/mL at 25°C, including that of apolipophorin 3 (figure 3).

**Figure 4:**
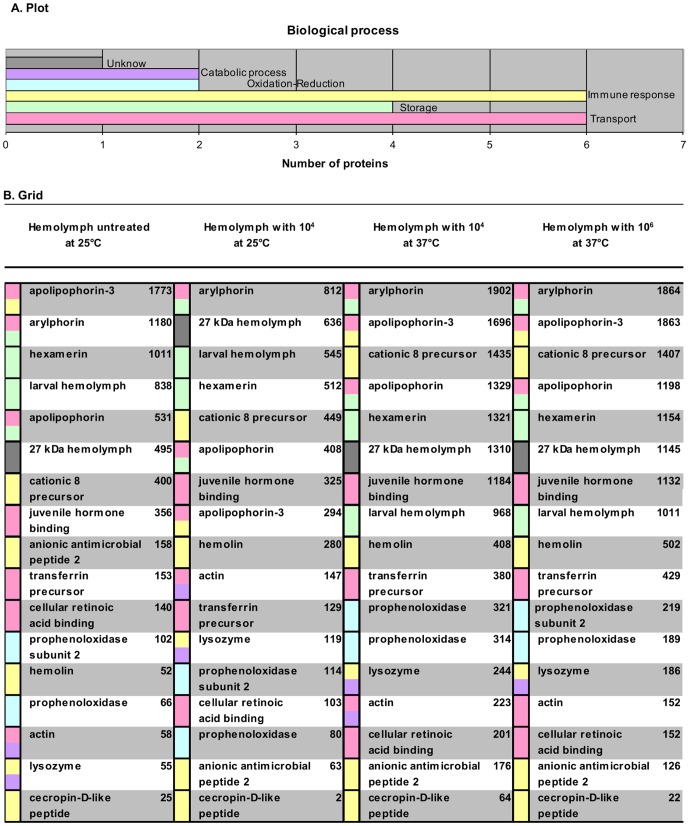
Biological process from 17 proteins. Colors in graph A and table B are related. A. Plot shows the number of proteins reported in databases in relationship with immune response. B. Grid represents either controls or microconidia concentrations in each treatment column (microconidia/mL). Column at the left of the protein name provides the color of the biological function. Some proteins have two or more functions, but for immune activity we selected two, including immune response. The column at the right of the protein name shows the concentration level of peptides. Gray represents an unknown function, but the protein domains were identified as follows: purple, catabolic process; blue, oxidation-reduction; yellow, immune response; green, storage; and red, transport.

Comparing the larvae at 10^4^ or 10^6^ microconidia/mL treatments, regardless of temperature, we observed two protein expression groups: one with high concentration levels (to the left of the graph) and the other with low concentration levels (to the right of the graph; figure 4). The first group included arylphorin, hexamerin, 27 kDa hemolymph, cationic 8 precursor, apolipophorin, juvenile hormone binding, apolipophorin 3. The second group included hemolin, transferrin precursor, prophenoloxidase subunit 2, lysozyme, actin, cellular retinoic acid binding, anionic antimicrobial peptide and cecropine D-like peptide.

Concerning the 10^6^ microconidia/mL treatment at 37°C, some of the proteins were slightly over-expressed compared with the 10^4^ treatment, such as the following: apolipophorin, juvenile hormone binding, apolipophorin 3, hemolin and transferrin precursor (these proteins are in the middle of the plot, figure 3). This pattern differs from the remaining proteins that never surpassed the 10^4^ microconidia/mL at the 37°C treatment.

Finally, we validated the results of expression on iTRAQ using RT-PCR, statistic correlations were found between the proteins analyzed for both techniques. Effectively, the genes selected: serpin, cationic protein 8p and 26 kDa ferritin subunit showed the same pattern of expression between the analyzed microconidia concentrations and temperatures. The p-values for the three proteins were greater than 0.05 indicating that both sets of data match, see data set in tables S6A, S6B, S6C, S6D, S6E, S6F and figures S1, S2.

### Bioinformatics analysis and gene ontology of lepidoteran proteins

The iTRAQ approach was useful for identifying, not only protein expression levels at different microconidia concentrations, but also new homologous proteins in *G. mellonella* due to this insect's lack of whole genome annotation. This approach was interesting because the ProteinPilot and Mascot Distiller are primarily designed to identify proteins in the own genome of the species considered. For example, of the selected 17 proteins, two were not annotated for *G. mellonella*: actin and cellular retinoic binding protein. Among the 40 lepidopteran proteins, we found 20 new ones that had not yet been annotated for *G. mellonella* (listed in table 2).

Our search in Uniprot, Pfam and KEGG allowed us to correlate the information with Gene Ontology and to associate the biological process with its immunological function. Figure 4 shows a plot (A) with the results of this search. We defined five biological functions for 17 proteins, supported by bioinformatics information metadata on the Gene Ontology data-base: transport, immune response, storage, oxide-reduction and catabolism. Of those 17 proteins, 6 are specialized in transport; 6 carry out immunological responses; 4 work in storage; 2 operate in oxide-reduction; and 2 more serve in catabolism. One protein appeared to possess an unknown function: the 27 kDa hemolymph protein precursor (figure 4A and 4B). Apolipophorin, arylphorin, lysozyme and actin are all essential proteins that have been quite well studied. Furthermore, some of these and other proteins were previously reported to play major roles in immunological processes, such as apolipophorin, cationic 8 precursor, hemolin, anionic antimicrobial peptide 2, lysozyme, and cecropin D-like peptide. The role of prophenolxydase in the oxide-reduction process and the function of lysozyme and actin in the catabolic process are also well known. As discussed below, our results identified unknown proteins from *G. mellonella* that were most likely associated with transport, storage or immunological response challenges and affected by fungal microconidia invasion.

Further biological functions are also likely associated with immunological responses. However, the plot in figure 4A represents either one or two main functions of the 17 proteins studied, as related to the immunological defense in Lepidoptera larvae oriented towards fungal invasion. Figure 4B shows the biological process for every protein. Apolipophorin 3, arylphorin, apolipophorin, actin and lysozymes perform two functions that are likely associated with immunological defenses, as shown in our results. Conversely, hexamerin, larval hemolymph, cationic 8 precursor, juvenile hormone binding, transferrin precursor, cellular retinoic acid binding, prophenoloxydase subunit 3, hemolin, prophenoloxydase, and two peptides (anionic antimicrobial 2 and cecropin D-like) contribute to one known function related to immunological response. There is no information concerning the 27 kDa hemolymph in the existing databases (figure 4B).

### Hypothetical structure of unidentified proteins of *G. mellonella*

Two proteins have not been annotated in any protein data bank for *G. mellonella*: actin (actin 4) and cellular retinoic acid binding protein (lipocalin family). Those important orthologous proteins were inferred based on sequences of close Lepidoptera species, such as *B. mori* for actin 4 (GenBank: AGR44824.1 or AGR44827.1) and *D. plexippus* for cellular retinoic acid binding protein (GenBank: EHJ79039.1).

**Figure 5:**
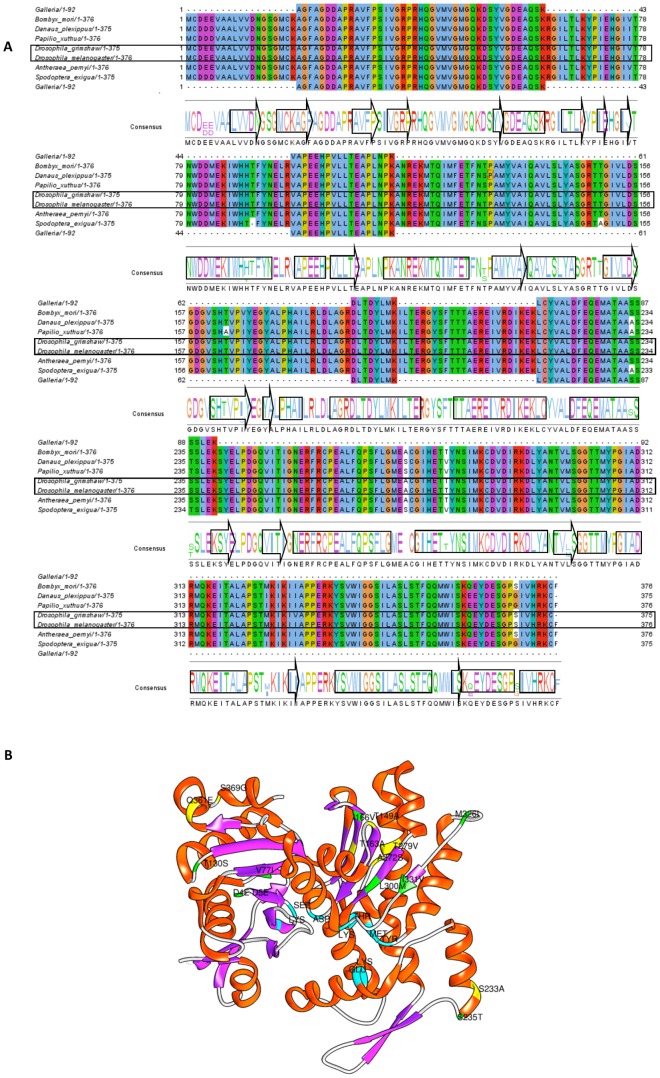
Actin 4 alignment and hypothetical 3D structure. A. The actin alignment of Lepidoptera protein sequences matches that of *G. mellonella*'s actin peptides (first discontinued sequences and dotted) and aligns well (100%) with the *B. mori* protein. Related species and *Drosophila* species were also included. A consensus was obtained on Jalview using a Clustal algorithm. The consensus sequence includes arrows (α helix) and cylinders (β-sheet) obtained by structural analysis. The alignment shows insertions, deletions or substitutions. B. Original tertiary 3MN6 (Protein Data Bank) actin pattern and probable amino acid substitutions are marked in yellow and green. The catalytic site residues can be found in blue.

The eight peptides of actin 4 identified by iTRAQ 8 plex from *G. mellonella* exactly match 100% of the *B. mori* actin 4 (GenBank: AGR44824.1, 376 AA), the *D. melanogaster* actin (GenBank: AAA28314.1 or NP_511052.1, 376 AA), the *D. grimshaw* (GenBank: XP_001986647, 375 AA) actin, and the *Antherae pernyi* actin (GenBank: ADJ67594, 376 AA). Those peptides also match 98.91% with the next Lepidoptera species due to their amino acid mutation in S233A and with *D. plexippus* (GenBank: EHJ70060), *S. exigua* (ADJ67594.1, 376 AA) and *P. xuthus* (GenBank: BAG30799) actin (figure 5A). The sequence homology between 376 AA from *B. mori* and *D. melanogaster* is 100%. The alignment with these actin sequences has shown that the conserved domains are the nucleotide-binding domain of the sugar kinase/HSP70/actin superfamily and that the catalytic site residues are exactly the same as the *D. plexippus* sequence (K18, D157, K213, E214, T303, M305, Y306, K336, and the highly conserved secondary and tertiary structure; figure 5A and 5B in blue). The consensus sequence takes the secondary structure hypothesized by the Phyre and i-Tasser servers. We also hypothesized a tertiary structure using the 3MN6 actin structure (Protein Data Bank) from *D. melanogaster* as a pattern, comparing it with AGR44824.1 actin 4 from *B. mori*. The probable substitutions are shown in yellow and green (figure 5B), with the following 18 possible amino acid substitutions in *G. mellonella*: T149A, T163A, S233A, A272S, T279V, Q361E, S369G, (in yellow) E4D, E5D, V77I, T130S, I166V, S235T L300M, M326I, I331V (in green), H89del, and F276del (figure 5B).

**Figure 6:**
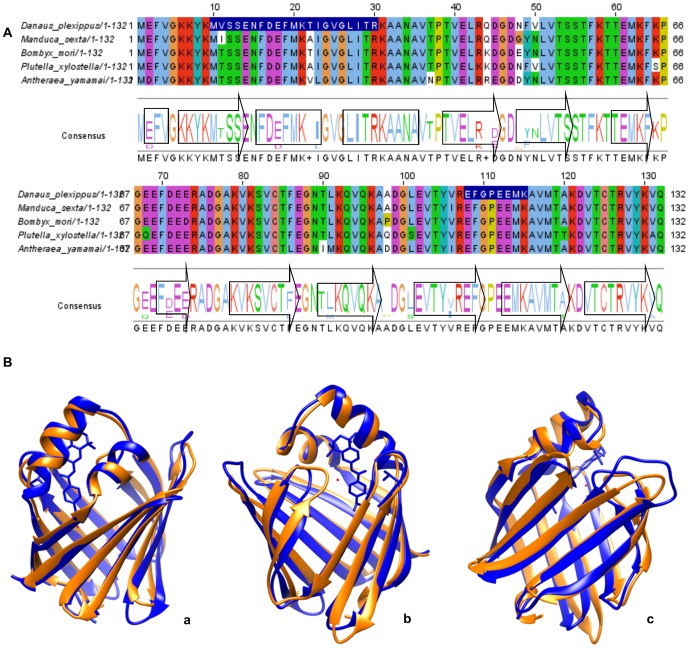
Cellular retinoic acid binding protein or lipocalin A. A. Alignment of different cellular retinoic acid binding proteins from five different Lepidoptera sequences. In the blue box, peptides identified by iTRAQ over the *D. plexippus* sequence can be found. A consensus was obtained on Jalview using Clustal algorithm, including the arrows (α- helix) and cylinders (β-sheet) obtained through a structural analysis. B. Three different side angles amid tertiary 2CBR (Protein Data Bank) cellular retinoic acid binding patterns with our hypothetical lipocalin structure. The alignment of the 3D structures (a,b,c) between the hypothetical *G. mellonella* CRABP (orange) and 2CBR (Protein Data Bank, in blue with ATP) cellular retinoic acid binding protein (136 AA) from *B. taurus*.

The three peptides of the cellular retinoic acid binding protein of *G. mellonella*, identified by iTRAQ 8 plex, match exactly 100% of the *D. plexippus* cellular retinoic acid binding protein (GenBank: EHJ79039.1, 132 AA). A lower percentage match was found for *M. sexta* cellular retinoic acid binding protein (GenBank: AAC24317.1, 132 AA), *B. mori* cellular retinoic acid binding protein (GenBank: NP_001037364.1, 132 AA), *Plutella xylostella* cellular retinoic acid binding protein (GenBank: BAD26694.1, 132 AA) and *A. yamamai* cellular retinoic acid binding protein (GenBank: AGG56524.1, 132 AA). The alignment with those cellular retinoic acid binding protein sequences showed the conserved domains between them (lipocalin superfamily) and their consistent conserved secondary structure (figure 6A). The consensus sequence takes the secondary structure hypothesized by Phyre and i-Tasser servers. The probable substitutions showed the 20 most likely amino acids to be replaced in *G. mellonella*: E2D, V11I/V11T, T22A/T22V, I23L, E28D, T37N, R43K, Q44K/Q44R, D45E, N48G/N48E/N48D, F49Y, V50N, K65S, E68Q, T90I, A98P/A98Q/A98D, L101S, V106I, A120T, and V131A. We also hypothesized a tertiary structure using the EHJ79039.1 cellular retinoic acid binding protein from *D. plexippus* on Phyre server as a pattern. The 3D alignment between the hypothetical structure (orange) and 2CBR (Protein Data Bank, in blue with ATP) cellular retinoic acid binding protein (136 AA) from *Bos taurus* was made using the Phyre server and visualized with UCSF Chimera version 1.8. This alignment is showing a structural homology with slightly structural differences, despite the low sequence homology with the *B. taurus* sequence (figure 6B).

## Discussion

### Immunological model and iTRAQ performance

*G. mellonella* larva is an excellent model to study biological process such as immunological responses and virulence mechanisms and the innate immune system. In our study, we infected the caterpillars with a *F. oxysporum* microconidia suspension. Their survival and mortality were statistically consistent. The hemolymph proteins were in high concentration, as noted by a Bradford assay.

The mold *F. oxysporum* is an inter-kingdom pathogen system and an emergent human, mammalian and vertebrate pathogen [37]. The very same mold strain might equally affect animals and plants [38]. We noted that the larvae of *G. mellonella* incubated at 37°C showed an enhanced humoral immune response after fungal infection compared with infected larvae incubated at 25°C. This enhanced response was manifested by an increased expression of proteins, as other authors have noted with yeast or molds [4], [39], [40], [41]. The larvae hemolymph is able to inhibit *Fusarium* filamentation and after 48 h can still resist from 10^4^ to 10^6^ microconidia/mL. The quantitation of iTRAQ is achieved by the comparison of peak areas and resultant peak ratios for eight LC/MS/MS reporter ions, ranging from 113–119 to 121 Da. In our case, 119 and 121 were not considered. The ratio of the signal intensities from these tags acts as an indicator of the relative proportion of that peptide between the differently labeled samples [42]. The minimal amount of protein identification achieved after iTRAQ 8 plex analyses was first attributed to the lack of genome information for *G. mellonella*. However, the strategy of matching peptides with closely related species allowed us to resolve and identify *G. mellonella* proteins in the immune response. Thus, it should be feasible to expect a large number of proteins to be identified by their similarity with well characterized insects, such as *Anopheles gambiae*, *Aedes aegypti*, *Apis mellifera* and *D. melanogaster*. These results seem to indicate that lepidopteran species could have evolved independently, a long time ago, from other insects or, at least, that the *G. mellonella* hemolymph proteins, related to the innate immune system, may be absent in other insect species.

### Protein expression induced by the fungal attack

Butterflies are holometabolous, meaning that pupae do not feed during metamorphosis; instead, they depend on nutrients that were previously accumulated in the larval period. Proteins, lipids and carbohydrates that have been selectively taken up by the larval fat body from the hemolymph before pupation [43] provide energy and amino acid building blocks for the imaginal tissues.

Banville *et al.* [44], found a down-regulated expression of a range of proteins associated with immune responses in a proteome analysis of 7-day starved larvae. In our case, we found the same result after a 2-day infection with *F. oxysporum* at 25°C, in contrast with a remarkable proteome over-expression at 37°C. The 48 hours of starvation with infection challenge triggered the immune response rapidly, increasing temperature from 25 to 37°C, enhancing protein expression linked to the immune response. Unlike the larvae in the starved stage, which reduced their expression in a range of antimicrobial and immune proteins, including apolipophorin and arylphorin [44], these proteins had a remarkably increased expression at 37°C.

In our study, arylphorin (AAA74229), which is possibly a hexamerin, had the best peptide coverage and was likely the most abundant protein in the hemolymph, together with apolipophorins. These data were found because arylphorin was down-regulated at 25°C, but markedly up-regulated at 37°C at the same microconidial concentration. Apolipophorin (ALP, AAT76806) is a lipoprotein that plays a role in lipid transport and is likely to be associated with antimicrobial activity because lipids (LPs) are secreted to prevent microbial invasions. Furthermore, the functional properties of lipophorin as a lipid carrier are well-characterized [45]. Recently, the study by Banville *et al.* [44] demonstrated the reduced expression of a range of antimicrobial peptides that are down-regulated in association with the immune response.

Apolipophorin III (apoLPIII, P80703) was the most down-regulated protein in this study, especially after 10^4^ microconidia/mL at 25°C, but it was slightly up-regulated at 10^6^ microconidia/mL (not significant). This protein seems to be unmodified under physiological conditions and all functions and effects observed must be inherent to the protein's structure [46]. Mammalian apolipoproteins, such as apolipoprotein E (apoE), are involved in lipopolisacharide (LPS) detoxification, phagocytosis, and, potentially, pattern recognition. The apoLp-III insect protein is homologous to apoE [47]. ApoLpIII stimulates increases in the hemolymph antibacterial activity [48] and super-oxide production by the blood cells (hemocytes) [49]. The protein was found to participate in immune reactions as an LPS-binding protein (LBP) or as a potentiator of bacteriolytic activity in the hemolymph, but was not involved in lipid transport functions [50], [51]. We believe it probable that ApoLpIII, stimulates antimycotic activity in the hemolymph.

The 27 kDa hemolymph protein precursor (P27k, P83632) has been poorly studied, and very little is known about it in Lepidoptera. Its sister protein in the hemolymph, Spz C-106, controls the expression of the antifungal peptide Drosomycin (Drs) by acting as a ligand of Told1 and inducing an intracellular signaling pathway [52]. In our study, this protein is evidently present in the hemolymph before infection and is up-regulated even at 25°C. However, its expression was remarkably increased at 37°C. These results are striking because, based on our previous meta-analysis, this remarkable expression was unexpected. Richards and Edwards [53], detected the 27 kDa hemolymph protein after 3 days of parasitization. Our results indicate that microconidial invasion stimulates this expression after 2 days.

Cationic protein 8 precursor (CP8, ADI87454) was an abundant protein that was markedly up-regulated at 25°C or 37°C in response to mycotic infection. This protein is a multiligand receptor that can recognize three microorganisms selected in other studies: *Escherichia coli*, *Micrococcus luteus*, and *C. albicans* [54]. The authors attempted to ascertain which immune responses were caused by the binding of the protein to these microorganisms. According to their results, CP8 in *G. mellonella* presented opsonin activity for the phagocytosis of the three microorganisms by hemocytes [54]. The iTRAQ data provided by this protein showed an extraordinarily up-regulated expression with microconidia invasion, regardless of the temperature.

Larval hemolymph protein (LHP, ABG91580) detected by iTRAQ, showed that this glycoprotein is down-regulated at 25°C, but up-regulated at 37°C in response to the infection. This protein is considered another storage glycoprotein that can have different degrees of glycosylation, including different patterns and molecular sizes (LHP74, 76, 76) 81, 82 and 84 [55]. The expression of the two Lhp genes of known sequences (Lhp76 and Lhp82) were monitored in both diapausing and non-diapausing *G. mellonella* individuals [56], [57]. However, those exceptional studies were not specific for immune responses, and at present, this protein is poorly investigated in *G. mellonella*, as opposed to past decades during which the protein was profusely studied [55], [58], [59].

Hexamerin (AAA19801) was another abundant protein in the *G. mellonella* results for this microconidia infection, as the protein is down-regulated at 25°C and up-regulated at 37°C in response to the infection. Storage hexamerins are important members of the hemocyanin superfamily. Although insects have storage hexamerins, very little is known about their characters or specific functions in *G. mellonella* and in general in insects [60]. Hexamerins are storage proteins with primordial functions in insect metamorphosis, but they may also have functions outside their role in storage, potentially even in immune responses [61].

Juvenile hormone binding protein (JHBP, AAN06604) is one of the main regulators of insect development and reproduction. It is crucial for proper insect development, acting as a transporter, protector and reservoir of the highly hydrophobic and chemically labile juvenile hormone, JH [62]. The expression was demonstrated to be maintained at a constant level when *G. mellonella* larvae were challenged with β-glucan [63]. JHBP has been found to down-regulate transferrin [64] and to bind to apolipophorin, arylphorin and hexamerin in the hemolymph, each of which are involved in JHBP molecular traffic [65]. In our study, JHBP maintained almost the same concentration at 25°C after microconidia infection, however, it was over-expressed or up-regulated at 37°C. Those results are in concordance with our findings, as JHBP was likely interacting with arylphorin, apolipophorin, hexamerin (high concentration) and transferrin precursors.

Transferrin has the ability to remove essential iron ions to withstand pathogen invasion, thereby retarding microbial colonization of the insect's hemocoel [66]. Banville *et al.* [44], found that ferritin and transferrin precursor were reduced in expression by approximately 50% in food-deprived larvae. In our study, the transferrin precursor (AAQ63970) was down-regulated at 25°C, but up-regulated at 37°C. Seitz, *et al.* [66] found genes that showed homologies with molecules, such as transferrin, which is known to be involved in immunomodulation after bacterial infection in *G. mellonella*. Transferrin, which is well known for its role in removing iron ions in vertebrates and thereby creating unfavorable environments for bacteria [67], might also be involved in an antibacterial iron-withholding strategies in insects [68]. Transferrin is likely involved in immunomodulation after mycotic infection to create an unfavorable environment in the hemolymph for fungal invasion because the removal of iron is very important for pathogens. Transferrin acts as an antimycotic mediator, preventing oxidative stress and enhancing survival against infections.

Hemolin (ACU09501), a member of the immunoglobulin protein superfamily, interacts in Lepidoptera as an opsonin, defending against potential pathogens and potentially playing a role in tissue morphogenesis. Hemolin also seems to be involved in insect defense against viral infections [69], but does not exhibit direct antibacterial activity. Instead, because it could be up-regulated 18-fold following bacterial injection in *Hyalophora cecropia* pupae and 30- to 40-fold in *M. sexta* larvae [70], this protein is thought to be the major inducible protein against fungi in the hemolymph. In our case, we found similar results because the protein was up-regulated 5- to 10-fold with *F. oxysporum* from 10^4^ to 10^6^ microconidia/mL at 37°C. The mechanism includes viral interaction with the hemolin anchored to the cellular membrane of the hemocytes. Because hemolin is associated with the hemocytes [71], [72], its function as an opsonin facilitates pathogen recognition and mediates hemocytic immune responses [73], [74]. We consider hemolin to have shown important activity in antimycotic immune responses, as its expression was always up-regulated when temperature or microconidia concentration were elevated.

We also found that prophenoloxidase (POO, AAK64363) and prophenoloxidase subunit 2 (PPOs2, AAQ75026) are important proteins in immune responses. These proteins make up the other coupled group found in this study, similar to apolipophorins and hexamerins. In general, phenoloxidase occurs in arthropod's hemolymph or hemocytes in its inactive form as a proenzyme called prophenoloxidase (PPO) [75], [76]. The activation of the prophenoloxidase triggers the enzymatic reaction that synthetize cytotoxic quinones. The latter protein can be toxic to the host tissue if it is not regulated to the site of infection [5]. The protein has been studied after conidia invasion. The sera from those larvae that received an injection of 10^5^ conidia of *A. fumigatus* showed the greater binding of PPO to the conidia compared with the sera from control larvae; however, all pre-treatments resulted in higher levels of this protein's binding [77]. There was evidence of an interaction between the coagulation system and the prophenoloxidase activating cascade [78].

A major defense in the innate immune system in invertebrates is melanization in response to pathogens or damaged tissues. This process constitutes the first humoral defense and is controlled by the enzyme phenoloxidase (PO) [79]. The proPO-activating system is triggered by the presence of minute amounts of compounds of microbial origins, such as β-1,3-glucans, lipopolysaccharides, and peptidoglycans, ensuring that the system will become active in the presence of potential pathogens [79]. We found that PPO and PPO subunit 2 proteins had the same patterns of expression in different treatments and remained at the same concentration at 25°C with or without microconidia. However, these proteins were significantly up-regulated with *F. oxysporum* from 10^4^ to 10^6^ microconidia/mL at 37°C.

Lysozymes (P82174) are most likely the best studied antibacterial enzymes and act as a model for structure-function studies [80]. However, these proteases are poorly studied in insects' innate immune systems. The insect's immune response is mediated by pattern recognition proteins that signal to expression effectors, including the antibacterial lysozyme protein [81], [82]. We found that lysozymes comprise important antimycotic enzymatic activities, as noted by its antiviral or antibacterial activity in many *in vitro* studies. We observed the remarkable over-expression of lysozymes in all microconidia treatments in comparison to the controls. It appears that lysozymes take on other roles besides degrading the bacterial cell wall, and it has been shown that this protease inhibits the enzymatic activity of mosquito phenoloxidase, thereby modulating melanization [82], [83].

Recently, the study by Banville *et al.* [44] demonstrated the reduced expression of a range of antimicrobial peptides that are down-regulated in the expression of a range of proteins associated with the immune response. We found it an interesting coincidence that cecropin-D-like peptide (P85210) and anionic antimicrobial peptide 2 (P85216) were both present in low concentrations. iTRAQ is likely the most accurate technique for studying peptides. Cecropins might normally offer protection against bacteria, but we found a marked down-regulation of Cecropin-D-like and Anionic antimicrobial peptide 2 peptides. In larvae challenged with fungi, these peptides were slightly up-regulated at 37°C compared with controls and other proteins. It is possible that these peptides are important among insects' immune responses to bacteria, according to Mak *et al.* [84], but are irrelevant for immune responses to fungi. Other studies should be undertaken to better clarify their final role *in vivo*, as these peptides have been well studied *in vitro* [28], [85], [86]. An interesting transcriptome study by Vogel *et al.* [87] catalogued the immune responses of a large number of peptides and proteins to lipopolysaccharides. In our study with fungi, we found a low expression of the serine protease inhibitor; however, we believe that it is still difficult to correlate immune responses between the transcriptome and proteome with different antigens.

### Expression in the immune response and structure of two renowned orthologous proteins

Unlike the most recent proteins discussed, actin (AGR44824 from *B. mori*) and cellular retinoic acid binding protein (CRABP, lipocallin family) are quite well studied in eukaryotic organisms. Although, they still have not been annotated in *G. mellonella* and are associated with many biological processes including immune responses. In this study, the actin expression was up-regulated continuously at any microconidia concentration or any set temperature. This result is in accordance with those of other authors because actin-5C, one of two cytoskeletal proteins, is up-regulated after short H_2_O_2_ treatments or other stress situations, such as anoxia or ethanol treatment in *D*. *melanogaster* [88]. The functional genomic analysis of phagocytosis also established the participation of actin cytoskeleton regulation proteins in innate immunity of *D*. *melanogaster* [89]. During the course of an infection, immune cells migrate toward the site of microbial entry with the goal of eliminating pathogens. This process requires not only cell migration but also changes in cell adhesion and phagocytosis, which universally depend on the dynamics of the actin network (i.e., the polymerization or depolymerization of actin filaments) [90].

Cellular retinoic acid binding protein (CRABP, EHJ79039 *D. plexippus*) is an important carrier of retinoic acid (RA or vitamin A) and, in mammals, is involved in inflammation and detoxification. RA is a potential chemotherapeutic agent in the treatment of cancers [91], [92] and is critical as a regulator of proper skin function [93]. There are few studies on CRABP and the innate immune system in insects; however, various works have characterized the protein in other organisms [94], [95]. This is an important matter in mammals because RA is a key modulator of the innate immune system [96]. As in other proteins in our study, CRABP was down-regulated with 10^4^ microconidia/mL at 25°C, but up-regulated at 37°C (non-significant). CRABP either was not greatly affected or produced similar results to the controls at 10^6^ microconidia/mL at 37°C, showing that CRABP most likely transports RA and lipids during stress conditions to stimulate the immune system.

The iTRAQ approach was not only useful to detect and quantify peptides; it was also useful to determine protein structures. It was interesting to address a 3D structural analysis using sequence peptides because the homologous protein information in closed species has been well supported, and the protein structure has been thoroughly investigated. Actin has been studied in insects [97] and three-dimensional structures have been characterized in *D. melanogaster*, such as the 3MN6 actin structure at the Protein Data Bank [98]. Compared with other lepidopteran or insect actins, our peptides are significantly conserved, allowing the inference of the actin's secondary or tertiary structure. Even if the *G. mellonella* actin structure is slightly different from *D. mellanogaster* actin, the homology of 7 peptides with *D. plexippous* actin allows the study of such structures in Lepidoptera. Non-synonymous substitutions do not seem to change the basic structure of the insect, as determined with *D. mellanogaster* 3MN6. One deletion was present only in the last AA C-terminus, and the number of hypothetical α-helices and β-sheets did not change domains.

The structure of CRABP has been well-studied in humans [99], and three-dimensional structures have been examined in 2CBR CRABP cow structure (Protein Data Bank). The structural homology of CRABPs between insects and mammals is considerable, reflecting their conserved functions among animals [99]. In *M. sexta*, the protein structure noticeably compared with those of mice and cows, as in our case [94]. Non-synonymous substitutions did not change the basic structure or domains. Finally, the number of hypothetical α-helices and β-sheets was a little shorter or longer than that in the 2CBR CRABP cow protein.

## Conclusions

As in other works, our study verified that the use of isobaric tags for the relative and absolute quantification (iTRAQ) of protein expression is a powerful proteomic tool that can be combined with the accuracy of Mass Spectrometry for protein identification. This tool constitutes a consistent method to detect proteins associated with the innate immune system of *G. mellonella* in response to infection caused by *F. oxysporum*.

In addition, iTRAQ was a reliable quantitative proteomic approach to detect and quantify the expression levels of immune system proteins and peptides, showing differential expressions in varying temperatures and microconidia concentrations, especially when the larvae were resistant to microconidia treatment. Our proteomic study allowed us to monitor the improvement of the immune response of *G. mellonella* against challenges with *F. oxysporum* at 37°C. In particular, it was found that 10^4^ microconidia/mL at 37°C over expressed many more proteins than other treatments. It is likely that most of the 17 main proteins contributed to caterpillar survival by protecting against the fungal infection and taking advantage of the heat shock, generating a real benefit in terms of evolutionary fitness.

The statistical validation of protein expression by RT-PCR allowed us to infer that iTRAQ expression data was consistent. It is likely that all proteins expressed in stress conditions (microconidia infection and high temperature) have not only been due to the exclusive effect of immunological response because some proteins were stimulated by temperature or metamorphic grown. In our case, infection and temperature increase were the main causes to over-express many of the 17 proteins studied, as supported by bibliographic metadata, but it is necessary to deepen in the *G. mellonella*-*F. oxysporum* model to better understand the innate immune system of Lepidoptera.

## Supporting Information

Figure S1 **Data from table S6D.** Sets of validation iTRAQ results. Validation of iTRAQ results with q-PCR. The S6 sets of tables (A, B, C, D, E and F) and Figures S1 and S2 results.
(DOCX)

Figure S2 **Data from table S6E.** Sets of validation iTRAQ results. Validation of iTRAQ results with q-PCR. The S6 sets of tables (A, B, C, D, E and F) and Figures S1 and S2 results.
(DOCX)

Table S1 **Bradford assay.** Bradford quantification of proteins in hemolymph after caterpillar sacrifice.
(DOCX)

Table S2 **F and t tests for 10^4^, 10^5^, 10^6^ microconidia/mL at 25 and 37°C.** First statistical assessment for microconidia concentration at 25 and 37°C. The concentrations 10^7^ and 10^8^ displayed sn (no survivals) and nt (not tested).
(DOCX)

Table S3 **F and t tests for 10^4^, 10^6^ microconidia/mL at 25 and 37°C.** Second statistical assessment for 10^4^ and 10^6^ microconidia/mL concentration at 25 and 37°C.
(DOCX)

Table S4 **The 59 proteins detected by iTRAQ.** ITRAQ Result: S1–S4, C1–C4 bias ProGroup NCBInr. Denominator 113, from YEPD repository platform.
(DOCX)

Table S5 **The 17 proteins detected by iTRAQ.**
(DOCX)

Table S6 **Sets of validation iTRAQ results.** Validation of iTRAQ results with q-PCR. The S6 sets of tables (A, B, C, D, E and F) and Figures S1 and S2 results. **Table S6A**, Pair of channels selected randomly. Next Data (tables and plots) are the statistic assessment to evaluate the relationship between iTRAQ and q-PCR. **Table S6B**, iTRAQ protein ratio relationship. **Table S6C**, Q-PCR from mRNA. **Table S6D**, Comparison iTRAQ-Q-PCR. **Table S6E**, Comparison of Q-PCR-iTRAQ. **Table S6F**, Chi-square for iTRAQ-Q-PCR.
(DOCX)
