# Supplementary material for: Development of Quantitative Proteomics Using iTRAQ Based on the Immunological Response of Galleria mellonella Larvae Challenged with Fusarium oxysporum Microconidia
Source: PLoS One. 2014 Nov 7;9(11):e112179. doi: 10.1371/journal.pone.0112179 (PMC4224417; doi:10.1371/journal.pone.0112179)
Supplement: Figure S1 — Data from table S6D. Sets of validation iTRAQ results. Validation of iTRAQ results with q-PCR. The S6 sets of tables (A, B, C, D, E and F) and Figures S1 and S2 results. (DOCX) [file pone.0112179.s001.docx]

**Figure S1.** Data from table S6D
