# Supplementary material for: Development of Quantitative Proteomics Using iTRAQ Based on the Immunological Response of Galleria mellonella Larvae Challenged with Fusarium oxysporum Microconidia
Source: PLoS One. 2014 Nov 7;9(11):e112179. doi: 10.1371/journal.pone.0112179 (PMC4224417; doi:10.1371/journal.pone.0112179)
Supplement: Table S1 — Bradford assay. Bradford quantification of proteins in hemolymph after caterpillar sacrifice. (DOCX) [file pone.0112179.s003.docx]

**Table S1.** **Bradford assay.** Bradford quantification of proteins in hemolymph after caterpillar sacrifice.

| **Sample** | **Absorbance** | **Protein concentration µg/mL** |
| --- | --- | --- |
| AM100m | 1.018 | 2500 |
| BT101p | 1.371 | 3461 |
| CR102y | 0.948 | 2310 |
| FM103x | 0.912 | 2212 |
| HK104u | 1.111 | 2753 |
| WH107c | 0.899 | 2176 |
