# Supplementary material for: Development of Quantitative Proteomics Using iTRAQ Based on the Immunological Response of Galleria mellonella Larvae Challenged with Fusarium oxysporum Microconidia
Source: PLoS One. 2014 Nov 7;9(11):e112179. doi: 10.1371/journal.pone.0112179 (PMC4224417; doi:10.1371/journal.pone.0112179)
Supplement: Table S2 — F and t tests for 104, 105, 106 microconidia/mL at 25 and 37°C. First statistical assessment for microconidia concentration at 25 and 37°C. The concentrations 107 and 108 displayed sn (no survivals) and nt (not tested). (DOCX) [file pone.0112179.s004.docx]

**Table S2**. **F and t tests for 10^4^, 10^5^, 10^6^ microconidia/mL at 25 and 37^o^C.** First statistical assessment for microconidia concentration at 25 and 37^o^C. The concentrations 10^7^ and 10^8^ displayed sn (no survivals) and nt (not tested).

|  | **Mean Survival** | | **F-test** | **t-test** | | |
| --- | --- | --- | --- | --- | --- | --- |
| **Treatment**  **microconidia/mL** | **25°C** | **37°C** | **Equivalent Value?** | **T** | **df** | **p-value** |
| 10^4^ | 9.23 | 9.90 | No | -2.8972 | 36 | < 0.01 |
| 10^5^ | 1.90 | 8.87 | Yes | -17.7242 | 43 | < 0.01 |
| 10^6^ | 0.47 | 8.87 | No | -38.7258 | 41 | < 0.01 |
| 10^7^ | ns | | nt | nt | nt | nt |
| 10^8^ | ns | | nt | nt | nt | nt |
