# Supplementary material for: Development of Quantitative Proteomics Using iTRAQ Based on the Immunological Response of Galleria mellonella Larvae Challenged with Fusarium oxysporum Microconidia
Source: PLoS One. 2014 Nov 7;9(11):e112179. doi: 10.1371/journal.pone.0112179 (PMC4224417; doi:10.1371/journal.pone.0112179)
Supplement: Table S3 — F and t tests for 104, 106 microconidia/mL at 25 and 37°C. Second statistical assessment for 104 and 106 microconidia/mL concentration at 25 and 37°C. (DOCX) [file pone.0112179.s005.docx]

**Table S3. F and t tests for 10^4^, 10^6^ microconidia/mL at 25 and 37^o^C.** Second statistical assessment for 10^4^ and 10^6^ microconidia/mL concentration at 25 and 37^o^C.

|  | **Mean Survival** | | **F-test** | **t-test** | | |
| --- | --- | --- | --- | --- | --- | --- |
| **Treatment microconidia/mL** | **25°C** | **37°C** | **Equivalent Value?** | **t** | **df** | **p-value** |
| 10^4^ | 9,1 | 10,0 | Yes | -3,1812 | 23 | < 0.01 |
| 10^6^ | 0,3 | 9,4 | Yes | -24,5798 | 21 | < 0.01 |
