# Supplementary material for: Development of Quantitative Proteomics Using iTRAQ Based on the Immunological Response of Galleria mellonella Larvae Challenged with Fusarium oxysporum Microconidia
Source: PLoS One. 2014 Nov 7;9(11):e112179. doi: 10.1371/journal.pone.0112179 (PMC4224417; doi:10.1371/journal.pone.0112179)
Supplement: Table S4 — The 59 proteins detected by iTRAQ. ITRAQ Result: S1–S4, C1–C4 bias ProGroup NCBInr. Denominator 113, from YEPD repository platform. (DOCX) [file pone.0112179.s006.docx]

**Table S4. The 59 proteins detected by iTRAQ**. ITRAQ Result: S1-S4, C1-C4 bias ProGroup NCBInr. Denominator 113, from YEPD repository platform

| **#** | **Protein Name** | **# Distinct Peptides in Ratios** | **115:113 ratio** | **115:113 p-value** | **116:113 ratio** | **116:113**  **p-value** | **117:113 ratio** | **117:113 p-value** |
| --- | --- | --- | --- | --- | --- | --- | --- | --- |
| 1 | apolipophorin * | 89 | 28.760 | 0 | 13.638 | 0.0324 | 28.904 | 0 |
| 2 | arylphorin * | 48 | 22.121 | 0 | 15.220 | 0.0002 | 21.889 | 0 |
| 3 | transferrin precursor * | 31 | 31.365 | 0 | 12.052 | 0.1128 | 33.433 | 0 |
| 4 | hexamerin * | 22 | 23.154 | 0 | 19.563 | 0 | 20.690 | 0 |
| 5 | Apolipophorin-III * | 20 | 43.993 | 0 | 57.507 | 0.0074 | 58.118 | 0 |
| 6 | prophenoloxidase subunit 2 * | 11 | 26.193 | 0 | 0.8090 | 0.0854 | 16.966 | 0.0044 |
| 7 | 27 kDa hemolymph protein * | 12 | 22.328 | 0 | 0.7711 | 0.0279 | 18.156 | 0 |
| 8 | Hemolin * | 21 | 13.760 | 0.0002 | 0.2604 | 0.0018 | 18.345 | 0 |
| 9 | Prophenoloxidase * | 14 | 31.297 | 0 | 0.8777 | 0.7673 | 24.634 | 0 |
| 10 | Apolipophorins | 2 | 30.268 | 0.1076 | 10.448 | 0.9322 | 29.908 | 0.1405 |
| 11 | trypsinogen precursor | 3 | 45.299 | 0.0036 | 0.5609 | 0.2289 | 34.096 | 0.0076 |
| 12 | Actin * | 7 | 13.167 | 0.0388 | 0.4065 | 0.0164 | 11.827 | 0.3534 |
| 13 | juvenile hormone binding protein * | 8 | 29.940 | 0 | 12.217 | 0.3657 | 32.726 | 0 |
| 14 | Lysozyme * | 6 | 26.897 | 0.0008 | 0.4344 | 0.219 | 14.758 | 0.0158 |
| 15 | beta-1,3-glucan recognition protein precursor | 1 | 52.526 |  | 37.167 |  | 49.886 |  |
| 16 | hexamerin receptor | 6 | 28.451 | 0 | 13.006 | 0.0806 | 21.421 | 0.0005 |
| 17 | cationic protein 8 precursor * | 5 | 37.626 | 0.0002 | 0.8482 | 0.2614 | 31.270 | 0.0002 |
| 18 | larval hemolymph protein * | 5 | 22.447 | 0.0137 | 16.487 | 0.0202 | 17.529 | 0.0534 |
| 19 | Apolipophorin-IIIb | 1 | 40.192 |  | 58.606 |  | 48.611 |  |
| 20 | antennal esterase CXE5 | 1 | 22.080 |  | 13.164 |  | 23.481 |  |
| 21 | apolipophorin precursor protein | 2 | 31.073 | 0.0557 | 10.796 | 0.7451 | 33.067 | 0.0528 |
| 22 | 32 kDa ferritin subunit | 4 | 49.406 | 0.238 | 16.622 | 0.7562 | 45.814 | 0.2852 |
| 23 | cellular retinoic acid binding protein * | 3 | 22.493 | 0.1784 | 15.563 | 0.3519 | 15.951 | 0.2884 |
| 24 | imaginal disc growth factor 4 | 4 | 29.615 | 0.0033 | 11.666 | 0.6247 | 29.788 | 0.0022 |
| 25 | serum albumin precursor | 1 | 34.048 |  | 0.4571 |  | 91.571 |  |
| 26 | glyceraldehyde-3-phosphate dehydrogenase | 1 | 23.119 |  | 30.144 |  | 38.804 |  |
| 27 | Apolipophorin-II; Apolipophorin-I | 3 | 29.724 | 0.0107 | 12.796 | 0.3813 | 27.463 | 0.0625 |
| 28 | glyceraldehyde-3-phosphate dehydrogenase | 1 | 24.460 |  | 0.2596 |  | 18.402 |  |
| 29 | proline-rich protein | 2 | 14.218 | 0.558 | 0.4424 | 0.6937 | 0.9606 | 0.9465 |
| 30 | 26kDa ferritin subunit | 3 | 33.140 | 0.0379 | 10.651 | 0.8401 | 31.325 | 0.0107 |
| 31 | Cecropin-D-like peptide * | 1 | 238.851 |  | 95.931 |  | 83.781 |  |
| 32 | hexamerin 2 | 1 | 29.790 |  | 17.782 |  | 26.511 |  |
| 33 | moderately methionine rich storage protein | 2 | 27.999 | 0.0732 | 0.9761 | 0.8737 | 20.078 | 0.1077 |
| 34 | filamin-C isoform 2 | 2 | 35.305 | 0.194 | 11.613 | 0.3723 | 39.223 | 0.136 |
| 35 | zinc finger protein 227-like | 1 | 21.977 |  | 12.049 |  | 19.801 |  |
| 36 | masquerade-like serine proteinase | 3 | 22.731 | 0.0118 | 10.083 | 0.973 | 19.589 | 0.0326 |
| 37 | Anionic antimicrobial peptide 2 * | 1 | 27.906 |  | 25.084 |  | 20.052 |  |
| 38 | moderately methionine rich storage protein | 2 | 25.236 | 0.2038 | 0.9465 | 0.9387 | 20.191 | 0.499 |
| 39 | Pyruvate kinase isozyme M1 | 1 | 13.032 |  | 0.3196 |  | 12.406 |  |
| 40 | hypothetical protein PHYSODRAFT_359907 | 1 | 24.131 |  | 10.354 |  | 25.558 |  |
| 41 | serpin 1 | 1 | 18.585 |  | 0.8506 |  | 24.064 |  |
| 42 | endopeptidase subunit C8 | 2 | 28.262 | 0.2522 | 0.6643 | 0.4501 | 22.623 | 0.0913 |
| 43 | ATP synthase subunit alpha, mitochondrial-like | 1 | -10.000 |  | -10.000 |  | -10.000 |  |
| 44 | isocitrate dehydrogenase | 1 | 25.011 |  | 27.864 |  | 29.861 |  |
| 45 | conserved protein, unknown function | 1 | 30.887 |  | 10.290 |  | 21.819 |  |
| 46 | hypothetical protein a | 1 | 30.166 |  | 13.241 |  | 30.848 |  |
| 47 | hypothetical protein b | 1 | 27.093 |  | 29.179 |  | 19.571 |  |
| 48 | 60 kDa heat shock protein, mitochondrial isoform 2 | 1 | 12.165 |  | 0.4297 |  | 25.433 |  |
| 49 | hypothetical protein c | 1 | 372.090 |  | 250.819 |  | 409.944 |  |
| 50 | Capsule synthesis positive regulator acpB | 1 | 21.195 |  | 11.808 |  | 25.939 |  |
| 51 | Kunitz-type protease inhibitor precursor | 1 | 39.741 |  | 10.152 |  | 31.493 |  |
| 52 | seminal fluid protein | 1 | 29.525 |  | 14.531 |  | 16.142 |  |
| 53 | peptidoglycan recognition protein SA | 2 | 45.781 | 0.1978 | 0.6720 | 0.168 | 35.192 | 0.1357 |
| 54 | hypothetical protein d | 1 | 31.401 |  | 0.7254 |  | 25.613 |  |
| 55 | DNA-directed RNA polymerase subunit beta' | 1 | 30.152 |  | 12.744 |  | 31.823 |  |
| 56 | FAD/NAD-P-binding domain-containing protein | 1 | 39.953 |  | 23.830 |  | 46.648 |  |
| 57 | hypothetical protein e | 1 | 45.872 |  | 13.911 |  | 19.485 |  |
| 58 | hemolymph storage protein 1 | 1 | 24.031 |  | 15.102 |  | 17.806 |  |
| 59 | diapause associated protein 2 | 2 | 21.869 | 0.1114 | 21.481 | 0.1468 | 15.460 | 0.1422 |
