# Supplementary material for: Development of Quantitative Proteomics Using iTRAQ Based on the Immunological Response of Galleria mellonella Larvae Challenged with Fusarium oxysporum Microconidia
Source: PLoS One. 2014 Nov 7;9(11):e112179. doi: 10.1371/journal.pone.0112179 (PMC4224417; doi:10.1371/journal.pone.0112179)
Supplement: Table S5 — The 17 proteins detected by iTRAQ. (DOCX) [file pone.0112179.s007.docx]

**Table S5. The 17 proteins detected by iTRAQ.** Selection from S4 table corresponding to the main 17 proteins studied. Other information about those proteins can be found in table 1. In red, the high p-values

| **#** | **# on last table S4** | **Protein Name** | **# Distinct Peptides in Ratios** | **115:113 ratio** | **115:113 P value** | **116:113 ratio** | **116:113 P value** | **117:113 ratio** | **117:113 P value** |
| --- | --- | --- | --- | --- | --- | --- | --- | --- | --- |
| 1 | 6 | apolipophorin | 89 | 28.760 | 0 | 13.638 | 0.0324 | 28.904 | 0 |
| 2 | 1 | arylphorin | 48 | 22.121 | 0 | 15.220 | 0.0002 | 21.889 | 0 |
| 3 | 10 | transferrin precursor | 31 | 31.365 | 0 | 12.052 | 0.1128 | 33.433 | 0 |
| 4 | 4 | hexamerin | 22 | 23.154 | 0 | 19.563 | 0 | 20.690 | 0 |
| 5 | 8 | Apolipophorin-III | 20 | 43.993 | 0 | 57.507 | 0.0074 | 58.118 | 0 |
| 6 | 12 | prophenoloxidase subunit 2 | 11 | 26.193 | 0 | 0.8090 | 0.0854 | 16.966 | 0.0044 |
| 7 | 2 | 27 kDa hemolymph protein | 12 | 22.328 | 0 | 0.7711 | 0.0279 | 18.156 | 0 |
| 8 | 9 | Hemolin | 21 | 13.760 | 0.0002 | 0.2604 | 0.0018 | 18.345 | 0 |
| 9 | 15 | Prophenoloxidase | 14 | 31.297 | 0 | 0.8777 | 0.7673 | 24.634 | 0 |
| 10 | 14 | Actin | 7 | 13.167 | 0.0388 | 0.4065 | 0.0164 | 11.827 | 0.3534 |
| 11 | 7 | juvenile hormone binding protein | 8 | 29.940 | 0 | 12.217 | 0.3657 | 32.726 | 0 |
| 12 | 11 | Lysozyme | 6 | 26.897 | 0.0008 | 0.4344 | 0.219 | 14.758 | 0.0158 |
| 13 | 5 | cationic protein 8 precursor | 5 | 37.626 | 0.0002 | 0.8482 | 0.2614 | 31.270 | 0.0002 |
| 14 | 3 | larval hemolymph protein | 5 | 22.447 | 0.0137 | 16.487 | 0.0202 | 17.529 | 0.0534 |
| 15 | 13 | cellular retinoic acid binding protein | 3 | 22.493 | 0.1784 | 15.563 | 0.3519 | 15.951 | 0.2884 |
| 16 | 17 | Cecropin-D-like peptide | 1 | 238.851 |  | 95.931 |  | 83.781 |  |
| 17 | 16 | Anionic antimicrobial peptide 2 | 1 | 27.906 |  | 25.084 |  | 20.052 |  |
