# Supplementary material for: Development of Quantitative Proteomics Using iTRAQ Based on the Immunological Response of Galleria mellonella Larvae Challenged with Fusarium oxysporum Microconidia
Source: PLoS One. 2014 Nov 7;9(11):e112179. doi: 10.1371/journal.pone.0112179 (PMC4224417; doi:10.1371/journal.pone.0112179)
Supplement: Table S6 — Sets of validation iTRAQ results. Validation of iTRAQ results with q-PCR. The S6 sets of tables (A, B, C, D, E and F) and Figures S1 and S2 results. Table S6A, Pair of channels selected randomly. Next Data (tables and plots) are the statistic assessment to evaluate the relationship between iTRAQ and q-PCR. Table S6B, iTRAQ protein ratio relationship. Table S6C, Q-PCR from mRNA. Table S6D, Comparison iTRAQ-Q-PCR. Table S6E, Comparison of Q-PCR-iTRAQ. Table S6F, Chi-square for iTRAQ-Q-PCR. (DOCX) [file pone.0112179.s008.docx]

**Table S6 series and Figures S1-S2.** **Sets of validation iTRAQ results.** Validation of iTRAQ results with q-PCR. The S6 sets of tables (A, B, C, D, E and F) and Figures S1 and S2 results.

**Table S6A**. Pair of channels selected randomly. Next Data (tables and plots) are the statistic assessment to evaluate the relationship between iTRAQ and q-PCR.

| **iTRAQ channel** | **Treatment** |
| --- | --- |
| 117 | Hemolymph challenge with *F. oxysporum* 10^6^ microconidia/mL at 37 ºC |
| 118 | Hemolymph injected only with Tween-80 (0.1% v/v) at 37ºC |

**Table S6B**. iTRAQ protein ratio relationship

| **Protein** | **Ratio 117:118 (A)** | **Ratio 118:117 (B)** |
| --- | --- | --- |
| cationic protein 8 precursor [Galleria mellonella] | 1,1234 | 0,8901 |
| 26kDa ferritin subunit [Galleria mellonella] | 0,7439 | 1,3443 |
| serpin 1 [Danaus plexippus] | 1,4552 | 0,6872 |

**Table S6C.** Q-PCR from mRNA

| **Gene** | **117 treatment** | **118 treatment** |
| --- | --- | --- |
| CationicP8p *Galleria mellonella* | 1,1851 | 1 |
| 26 kDA Ferritin subunit *Galleria mellonella* | 0,4137 | 1 |
| Serpin 1 *Danaus plexippus* | 2,5112 | 1 |

**Table S6D.** Comparison iTRAQ-Q-PCR

| **Protein or gene** | **A/B** | **C/D** | **Difference** | **ratio** |
| --- | --- | --- | --- | --- |
| cationic protein 8 precursor *Galleria mellonella* | 1,2621 | 1,1851 | 0,0770 | 1,06 |
| 26kDa ferritin subunit *Galleria mellonella* | 0,5534 | 0,4137 | 0,1397 | 1,34 |
| serpin 1 *Danaus plexippus* | 2,1176 | 2,5112 | 0,3936 | 0,84 |

**Table S6E.** Comparison of Q-PCR-iTRAQ

| Protein or gene | **B/A** | **D/C** | **Difference** | **ratio** |
| --- | --- | --- | --- | --- |
| cationic protein 8 precursor *Galleria mellonella* | 0,792 | 0,844 | 0,0515 | 0,94 |
| 26kDa ferritin subunit *Galleria mellonella* | 1,807 | 2,417 | 0,6101 | 0,75 |
| serpin 1 *Danaus plexippus* | 0,472 | 0,398 | 0,0740 | 1,19 |

**Table S6F**. Chi-square for iTRAQ-Q-PCR

| **Protein or gene** | **Chi-Square** | **df** | **p-value** |
| --- | --- | --- | --- |
| cationic protein 8 precursor *Galleria mellonella* | 0.002 | 1 | 0.9646 |
| 26kDa ferritin subunit *Galleria mellonella* | 0.0408 | 1 | 0.8399 |
| serpin 1 *Danaus plexippus* | 0.0136 | 1 | 0.9072 |
